# Chromatin Immunoprecipitation to Analyze DNA Binding Sites of HMGA2

**DOI:** 10.1371/journal.pone.0018837

**Published:** 2011-04-14

**Authors:** Nina Winter, Rolf Nimzyk, Carolin Bösche, Anke Meyer, Jörn Bullerdiek

**Affiliations:** 1 Centre for Human Genetics, University of Bremen, Bremen, Germany; 2 Clinic for Small Animals and Research Cluster REBIRTH, University of Veterinary Medicine, Hannover, Germany; University of Illinois at Chicago, United States of America

## Abstract

**Background:**

*HMGA2* is an architectonic transcription factor abundantly expressed during embryonic and fetal development and it is associated with the progression of malignant tumors. The protein harbours three basically charged DNA binding domains and an acidic protein binding C-terminal domain. DNA binding induces changes of DNA conformation and hence results in global overall change of gene expression patterns. Recently, using a PCR-based SELEX (Systematic Evolution of Ligands by Exponential Enrichment) procedure two consensus sequences for HMGA2 binding have been identified.

**Methodology/Principal Findings:**

In this investigation chromatin immunoprecipitation (ChIP) experiments and bioinformatic methods were used to analyze if these binding sequences can be verified on chromatin of living cells as well.

**Conclusion:**

After quantification of HMGA2 protein in different cell lines the colon cancer derived cell line HCT116 was chosen for further ChIP experiments because of its 3.4-fold higher HMGA2 protein level. 49 DNA fragments were obtained by ChIP. These fragments containing HMGA2 binding sites have been analyzed for their AT-content, location in the human genome and similarities to sequences generated by a SELEX study. The sequences show a significantly higher AT-content than the average of the human genome. The artificially generated SELEX sequences and short BLAST alignments (11 and 12 bp) of the ChIP fragments from living cells show similarities in their organization. The flanking regions are AT-rich, whereas a lower conservation is present in the center of the sequences.

## Introduction

High mobility AT-hook 2 (HMGA2) is a chromatin-associated protein implicated in the development and progression of benign and malignant tumors as well as stem cell self-renewal [1], [2], [3]. Although some single target genes directly regulated by HMGA2 have been identified there is little doubt that it rather acts as a global chromatin switch than as a transcription factor controlling a few genes only [4], [5], [6]. On the other hand, its primary action as a chromatin-remodeling switch molecule requires a large number of DNA binding sites throughout the genome which would fit with its relative abundance e.g. in embryonic stem cells [7], [8]. Nevertheless, surprisingly little is known about possible patterns of its binding sites on the chromatin of living cells. Akin to the other mammalian HMGA proteins HMGA2 is characterized by three highly basic DNA-binding motifs called AT-hooks. All three AT-hooks show striking amino acid similarities with each other. Generally, the minor grooves of AT-rich DNA stretches are thought to represent suitable binding sites for the AT-hooks [9], [10]. Moreover, stable DNA binding apparently requires interacting of the central AT-hook and either of the two flanking hooks to DNA [11]. In a recent paper, Cui and Leng [12] have used a SELEX procedure for the analysis of the interactions of short random DNA fragments with HMGA2 to delineate consensus sequences for the binding of AT-hooks. The study has resulted in the identification of a DNA motif and its derivates strongly supporting HMGA2 binding but the results were obtained using naked DNA instead of chromatin fragments and comprehensive data on its chromatin binding in living cells are missing. Herein, we have performed a study based on chromatin immunoprecipitation (ChIP) from living cancer cells to analyze HMGA2 binding sites. The resulting fragments have been analyzed for a common binding motif as well as for similarities to the sequences emerging from the study by Cui and Leng [12].

## Results

### Isolation of HMGA2 binding sites via ChIP

**Figure 1:**
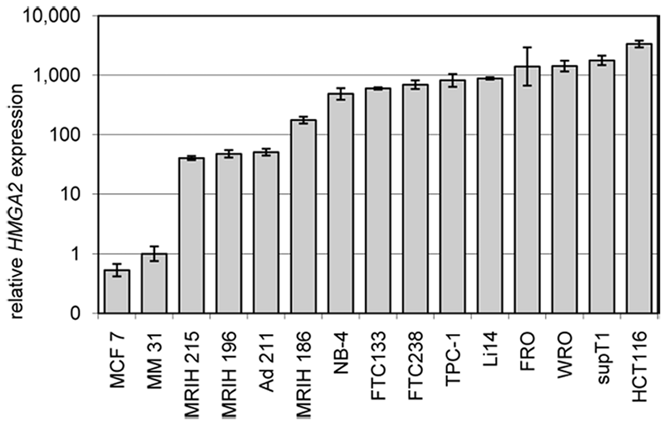
Relative expression of *HMGA2* in different samples. Origin of the various human cell lines and fresh sample: MCF 7 (mamma carcinoma), MM 31 (myometrium); MRI-H215, MRI-H196 and MRI-H186 (cervical carcinoma); Ad 211 (pleomorphic adenoma); NB-4 (promyelocytic leukemia); FTC133 and FTC238 (follicular thyroid carcinoma); TPC-1 (papillary thyroid carcinoma); Li14 (lipoma); FRO (anaplastic thyroid carcinoma); WRO (follicular carcinoma); supT1 (T cell lymphoblastic lymphoma); HCT116 (colon carcinoma).

**Figure 2:**
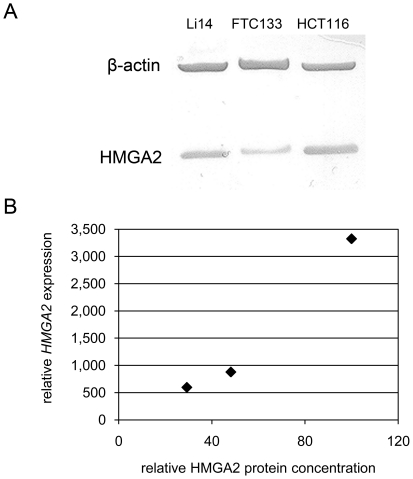
Western blot analysis of HMGA2 in different cell lines. (A) Expression of HMGA2 in three cell lines was determined using β-actin as endogenous control. (B) Comparison of *HMGA2* mRNA expression and HMGA2 protein expression in exemplary cell lines.

The first step for characterization of HMGA2 binding sites was to choose an adequate cell line showing high levels of HMGA2 for the following ChIP analyses. Therefore, we investigated the *HMGA2* mRNA expression of 14 cell lines and one tissue sample of human origin. RNA expression of cell line HCT116 (adenocarcinoma of the colon) was up to 3,300-fold elevated in comparison to sample MM 31 (myometrium). This expression of *HMGA2* mRNA in HCT116 was by far the highest among the cell lines investigated (Fig. 1). To check these results on the protein level Western Blot analysis was performed using selected cell lines. As shown in Figure 2 we detected HMGA2 in three cell lines and the amount of protein was calculated refering to β-actin as endogenous control. In the HCT116 cell line the relative amount of HMGA2 detected in Western Blot analysis was 2.1- (Li14) and 3.4-fold (FTC133) higher. This tendency corresponds to the relative *HMGA2* mRNA expression measured with real-time PCR, in which HCT116 shows a 3.8-fold (Li14) and 5.6- (FTC133) higher expression (Fig. 2B).

For this study two basic protocols for ChIP [13], [14] have been optimized for use with HCT116 cells. A flow diagram of the procedure is provided in Figure S1, and the details are given under materials and methods. The DNA enrichment within the samples was measured by real-time PCR and analysis was done by comparing the data from the immunoprecipitated sample (IP) against the background signal of the negative control without antibody (NoAb) to calculate the x-fold enrichment. In Table 1 the results of five samples used for ChIP followed by cloning of the DNA fragments are displayed. Average enrichment of the IP samples was 246-fold. The amplified gene sequence of GAPDH has no known HMGA2 binding site and served as a control to evaluate the DNA concentration and enrichment after ChIP.

**Table 1:** x-fold enrichment of the chromatin immunoprecipitated samples measured with *GAPDH* primers.

Sample	Average Ct-value	Ct^NoAb^-Ct^IP^	x-fold Enrichment
32 IP	29.85	8.55	374.29
32 NoAb	38.39		
33 IP	30.76	6.25	76.21
33 NoAb	37.01		
35 IP	30.37	8.86	464.65
35 NoAb	39.23		
36 IP	29.49	6.97	125.37
36 NoAb	36.46		
37 IP	29.84	7.57	190.15
37 NoAb	37.41		

**Figure 3:**
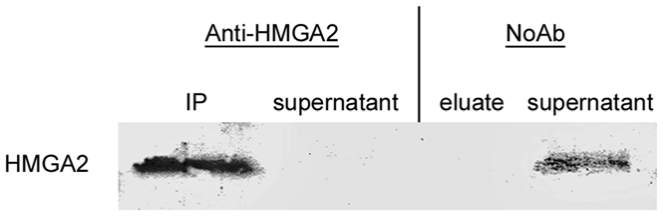
Western blot analysis of HMGA2 in ChIP samples. The analysis shows an enrichment of HMGA2 in the IP sample but not in the corresponding supernatant. No HMGA2 is detectable in the eluate of the NoAb control because HMGA2 remains in the supernatant of the non-immunoprecipitated sample.

Furthermore, the enrichment of HMGA2 during ChIP was confirmed by Western blot analysis (Fig. 3) as revealed by the presence of HMGA2 in the IP sample but not in the corresponding supernatant. In contrast, HMGA2 can be detected in the supernatant of the NoAb control and not in the eluate of the negative control. The immunoprecipitated DNA fragments were cloned into pGEM-T easy vector for blue/white screening. 49 clones from ChIP-derived DNA fragments were obtained and sequenced.

### Analysis of immunoprecipitated and sequenced DNA fragments

All 49 sequences were mapped to single loci in the human genome using the NCBI BLAST tool (Table 2). Length of ChIP DNA fragments ranged from 105 bp to 1848 bp with an average length of 517 bp. 23 of the cloned sequences were located intergenic and 23 intragenic. The remaining fragments matched to an unplaced genomic region not assigned to a chromosome until now. Table 2 displays the genes which are located upstream or downstream within the flanking regions of the 23 intergenic sequences, with a distance ranging from approximately 500 bp to 2000 kb. From a total of the 23 intragenic sequences seven were located within the first intron and three in the second intron. The remaining 12 sequences were assigned to various other introns, except for one fragment located in the second exon of a gene. Detailed sequences are listed in Table S1.

**Table 2:** 49 clones from ChIP-derived DNA fragments and their distribution in the human genome.

Clone	Length [bp]	AT [%]	Localization	Gene Symbol	Location to Gene	Distance to Gene
41	105	61	1p35	PTPRU	upstream	180 kb
28	403	68	1q25	SEC16B	downstream	70 kb
3	440	61	1q25.1	TNR	intron 1	-
23	253	63	1q31	KCNT2	downstream	1000 kb
14	652	69	1q31.1	FDPSL1	downstream	300 kb
27	1017	57	1q42	CDC42BPA	intron 21	-
8	715	68	2p13.3	GKN3P	intron 1	-
2	373	65	2p24.1	WDR35	intron 34	-
29	316	47	2q31	HOXD10	exon 2	-
48	1612	54	3p21	LARS2	intron 13	-
45	1592	59	3p22	STAC	downstream	170 kb
44	234	70	3q26.1	SI	downstream	25 kb
49	157	52	3q26.1	KPNA4	intron 1	-
40	171	62	4p15.1	ARAP2	downstream	2000 kb
16	395	63	4q31.1	CLGN	upstream	500 bp
12	981	69	4q32.3	SPOCK3	upstream	300 kb
10	350	73	4q34.3	RPL19P8	downstream	15 kb
39	561	60	5p14	PRDM9	downstream	117 kb
32	1080	61	6p22	DCDC2	intron 2	-
22	574	67	6q16	TSG1	upstream	113 kb
20	574	67	6q22	NKAIN2	intron 1	-
6	639	62	6q22.31	MAN1A1	downstream	830 kb
35	233	68	6q23	VNN3	upstream	900 bp
34	105	74	7q22	RELN	intron 33	-
17	323	58	7q36.1	ACTR3C	upstream	9 kb
4	492	72	8q21.12	PKIA	upstream	220 kb
7	219	72	8q23.2	PKHD1L1	intron 16	-
13	161	72	9q21.12	ALDH1A1	upstream	90 kb
36	276	52	9q22	COL15A1	intron 1	-
30	765	50	9q34	ENG	intron 8	-
46	180	69	10p11.2	CCDC7	downstream	32 kb
43	300	60	10p13	FAM107B	intron 2	-
15	305	71	10q21.3	JMJD1C	intron 22	-
5	1848	67	13q32.3	FGF-14	intron 1	-
11	363	71	14q21.3	RPL10L	downstream	350 kb
24	142	64	14q32	PPP4R4	intron 2	-
42	695	52	17q21	PLEKHM1	upstream	3 kb
33	1220	61	17q22	MBTD1	intron 6	-
19	508	51	17q23.3	RGS9	intron 19	-
18	495	64	18p11.22	PPP4R1	intron 23	-
31	412	60	18q21	STARD6	upstream	149 kb
26	287	64	19q12	ZNF99	downstream	25 kb
47	359	66	19q13.1	FCGBP	intron 3	-
9	511	66	20q13.11	PTPRT	intron 1	-
1	500	56	20q13.13	NFATC2	downstream	6.5 kb
25	235	46	21q22	RUNX1	intron 5	-
21	201	54	*			
37	633	62	*			
38	388	79	*			

*Unplaced genomic region.

For further analysis we compared our ChIP fragments with known binding sites, as predicted by Cui and Leng [12]. Two consensus sequences (5′-ATATTCGCGAWWATT-3′ and 5′-ATATTGCGCAWWATT-3′, where W represents A or T) have been described. The ChIP DNA sequences have been screened for the described consensus sequences, to analyze whether these putative binding sites are part of our ChIP sequences obtained herein. None of the consensus sequences was detected in the sequences revealed by ChIP. Next, the ChIP-generated fragments were compared with sequences containing lower constraint as identified by the SELEX [12] study. This lower constraint expanded the described HMGA2 binding sequences to 4,096 binding sites within the human genome. With a Perl program using a pattern match representing the 4,096 possibilities and NCBI BLAST standalone tool the sequences were scanned for possible HMGA2 binding sequences. Again, none of these putative HMGA2 binding sites was detected in sequences resulting from ChIP.

Because none of the sequences for HMGA2 binding described by Cui and Leng [12] matched within the isolated ChIP fragments, we scanned the human genome for the putative consensus sequences generated in the above mentioned SELEX study. For this the Perl program and the NCBI BLAST tool adapted to short sequences were used. Only six matches in the whole genome can be found for the conserved possible HMGA2 binding sites described by Cui and Leng [12] (NCBI refseq human genomic sequences build 36). If the consensus is extended to the 4,096 possibilities, 27,455 matches exist (Human genome NCBI refseq sequences Build 36). Thus, a possible binding site for HMGA2 would occur on average every 104,565 bp. In comparison to the theoretically expected occurrence of such a 15 bp sequence pattern (every 262,144 bp in the human genome) the consensus is 2.5 times more often attendant.

**Figure 4:**
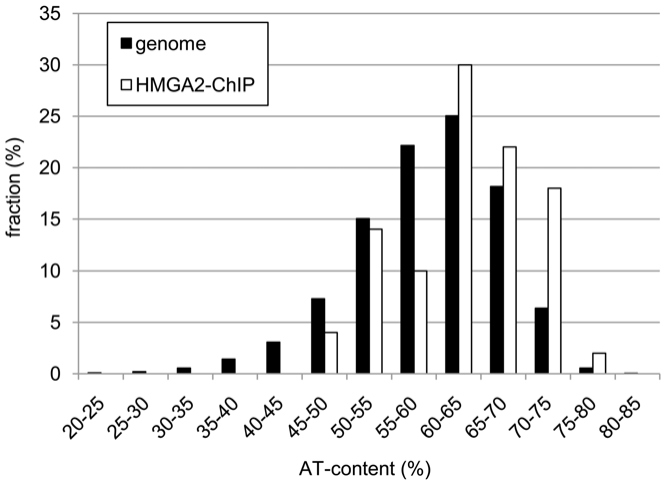
Histogram of the AT-content in the human genome and the ChIP DNA sequences. The whole human genome was split into pieces of 500 bp and AT-content was determined and compared to the AT-content of the sequences revealed by ChIP with HMGA2-antibody. The Wilcoxson rank sum test shows that the AT-content in the ChIP DNA sequences is significantly higher than in the human genom (p<0.0012).

Because HMGA2 is supposed to bind to the minor groove of AT-rich sequences [15], the sequences identified by the ChIP experiments have been analyzed for their AT-content. Approximately three-fourth of the sequences had AT-content exceeding the average of 59% in the human genome [16] with 20% of them even being highly AT-rich exceeding 70%. The AT-content in the deduced ChIP sequences was compared to the distribution in the human genome (NCBI refseq sequences Build 36), for that purpose the whole genome was split into pieces of 500 bp and the AT-content was determined (Fig. 4). For analysis of statistical significance and due to non-normality of the AT-distribution in the human genome [16] the Wilcoxson rank sum test as non-parametric alternative has been used. The distribution of the AT-content in the ChIP DNA sequences discribed herein is significantly higher than it would be expected in random fragments of the human genome (p<0.0012) (W = 105561580).

**Figure 5:**
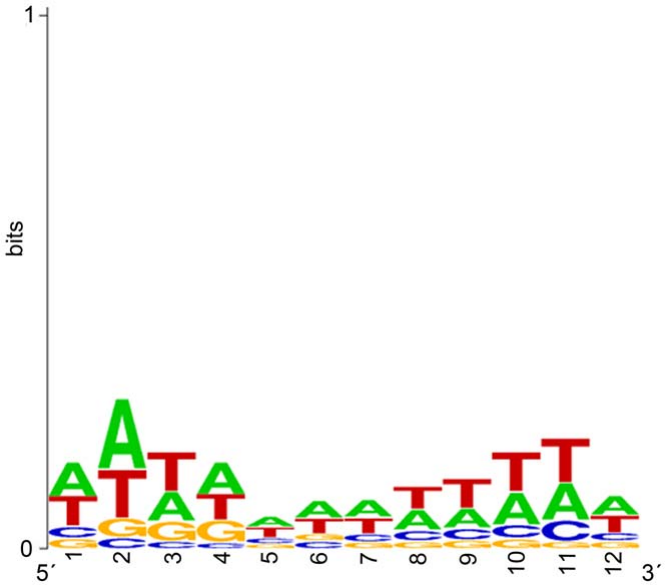
Sequence analysis of the concerted BLAST alignments of the detected ChIP sequences. The sequence logo was created by 12 bp long BLAST alignments. Sequence conservation, measured in bits of information, is illustrated by the height of stacking of the four letters for each position in the binding sites. The relative heights are proportional to their frequencies shown in the 134 BLAST sequences. The sequence logo was generated by WebLogo (available at http://weblogo.berkeley.edu/logo.cgi).

The cloned sequences were analyzed for the presence of any conserved sequences using the NCBI BLAST tool. This analysis shows a high rate of matches. These sequences have a significant higher AT-content compared to the human genome (W = 1169693292, p-value<2.2e^−16^, Wilcoxon rank sum test) and to the ChIP-isolated sequences themselves (W = 11787, p-value = 1.561e^−05^, Wilcoxon rank sum test). All sequences show multiple AT-stretches except for clone 25 and 49 containing only one AT-stretch. To identify further similarities between these BLAST matching sequences the 11 and 12 bp matches were adjusted manually from redundancies and used to create a sequence logo (WebLogo, http://weblogo.berkeley.edu/logo.cgi). In both cases the logo shows a higher AT-content in the flanking regions and no specificity in the middle of the sequence (Fig. 5).

## Discussion

A crucial question in field of gene regulation is where and to what extent transcription factors bind to DNA. This study is focused on the architectonic transcription factor HMGA2 which is abundantly expressed during embryonic and fetal development, whereas expression in normal fully differentiated adult cells is very low or even absent. This is the first time HMGA2 binding on chromatin in living cells is determined by ChIP analysis. The advantage of this method is that there is no need to prior identification of target genes regulated through binding of HMGA2. Furthermore, regulatory regions can be revealed wether they are located at promotors, introns or even distant enhancer elements.

In our study we selected a cell line with abundant expression of HMGA2 but this is not necessarily associated with malignant cellular behavior because, e.g. embryonic stem cells show a high level of HMGA2 associated with differentiation and cell proliferation during embryonic development [8]. Comparing the colon carcinoma cell line HCT116 with the thyroid carcinoma cell line FTC133 a drastical overexpression of HMGA2 both in the mRNA and the protein level compared to the myometrium was noted, the relationship between these two cell lines was in a comparable rang, i.e. HCT116 had a 5.6-fold higher expression of HMGA2 mRNA than FTC133 and a 3.4-fold higher expression on the protein level.

We compared the sequences of the DNA fragments obtained to results of a previously performed SELEX analysis on protein-free DNA [12]. These sequence motifs which should bind HMGA2 as described by Cui and Leng 2007 [12] have not been found in the DNA fragments revealed by ChIP. There are two possible explanations for the absence of corresponding sequences in the fragments identified by ChIP. First, HMGA2 proteins are not only interacting with DNA but also with a variety of other DNA binding and chromatin binding proteins like APEX1 (APEX nuclease (multifunctional DNA repair enzyme) 1) [17] and E4F1 (E4F transcription factor 1) [18]. If not distinguished from the highly similar HMGA1a and HMGA1b proteins, which is common in the literature, the HMGA proteins have a lot of molecular partners as transcription factors and other DNA binding proteins (reviewed in [19]). Considering this facts together with the direct involvement of HMGA2 in base excision repair with own enzymatic lyase activity [17] it is not likely that HMGA2 is binding DNA in a specific manner. Especially for the function in excision repair a specific binding site seems counterproductive to the occurrence of mutations only by chance not at specific sites.

The second possible explanation for the absence of similarities between the SELEX sequences and the ChIP DNA fragments is, that the occurrence of the consensus sequences for HMGA2 binding described by Cui and Leng [12] is rare and the statistics of appearance of a 15 bp sequence (approximately three times in the human genome, with the ambiguity approximately 18 times) implicates that these consensus sequences are of limited biological relevance for the HMGA2 activity. According to its function as a chromatin-remodelling switch HMGA2 is supposed to require a large number of DNA binding sites throughout the genome. This is consistent with the observations of relative abundance of HMGA2 in embryonic stem cells by Li *et al.* [7], [8]. Therefore, the rare occurrence of the consensus sequences has to be explained. Besides the possibility of artificial binding in the SELEX experiments the statistical 2.5 fold overrepresentation of the extended HMGA2 consensus sequences versus the representation of such sequences only by chance in the human genome points to a possible other explanation. The consensus motif described by Cui and Leng [12] is efficiently binding HMGA2 but *in vivo* this binding is maybe irrelevant. The binding might be too strong for purposes of dynamic regulation which is required for the proper activity of HMGA2.

The AT-content of the sequences generated by ChIP is significantly higher than the average of the human genome. This confirms the hypothesis that HMGA2 binds to AT-rich sequences. It therefore seems feasible to speculate that a motif with central GC bases and flanking AT bases is the possible target of HMGA2.

The analysis of the DNA fragments among each other shows a multitude of matches for conserved AT-stretches. All sequences but two contain multiple AT-stretches. A possible explanation for these two sequences having only one AT-stretch is that HMGA2 does not necessarily need DNA to interact with because it can bind to DNA- or chromatin binding proteins as well [17], [18]. To identify further similarities between these BLAST matching sequences, 11 bp and 12 bp matches were used exemplarily to create a sequence logo. Interestingly, the high AT-content in the flanking sequences resembles the pattern of the SELEX sequences presented by Cui and Leng [12]. This pattern has a central of 4 GC-rich bases flanked by AT-rich sequences. It is well known, that HMGA2 is a DNA binding protein that specifically recognizes the minor groove of AT-rich DNA sequences. One turn in DNA consists of approximately 10 bp and thus both presented patterns fit to the HMGA2 AT-hook composition and the winding of the DNA molecule.

HMGA2 is able to regulate certain genes via binding to promoter or enhancer regions, which are located upstream or downstream to the target gene, as well as intronic e.g. in case of the *IMP2* gene [20], [21]. Except for one sequence all possible binding sites were assigned to non-coding regions. Some of the genes identified to be located close to the generated ChIP DNA fragments play an important role in different types of cancer with high *HMGA2* expression. *RELN* and *ALDH1A1* are expressed in prostate cancer [22], [23], *ENG* [24], *SI* [25], *FCGBP* [26] and *PTPRT* [27] are associated with colonic tumors. *HOXD10* [28] and *MAN1A1* [29] show an up-regulated gene expression in breast cancer. The *RPL10L* gene is related to ovarian cancer [30] and *JMJD1C* plays an essential role in embryogenesis and carcinogenesis [31]. A functional relation between the oncofetal HMGA2 and the above mentioned genes is feasible and as a transcription factor HMGA2 is able to influence many different regulatory processes [32]. It remains to be elucidated, if HMGA2-binding is directly related to the up- or down-regulation of expression in this certain cases either through directly binding to DNA or in a complex with other proteins.

To the best of our knowledge this is the first approach to characterize possible HMGA2 binding sites in the chromatin of living cells by ChIP and cloning. Via protein-DNA binding HMGA2 plays important roles in tumor growth and stem cell-renewal. The possibility to screen, localize, and characterize the whole human genome for sequences bound to HMGA2, can help to understand in which way HMGA2 is associated with different biological processes.

## Materials and Methods

### Ethics Statement

The use of the human myometrium sample for this study was approved by the local medical ethics committee and followed the guidelines of the declaration of Helsinki. The patient gave written informed consent for clinical procedure and research use of the tissues.

### Cell culture

14 human cell lines and one sample of fresh tissue were examined in this study: MCF 7 (mamma carcinoma) [33], MM 31 (myometrium); MRI-H215, MRI-H196 and MRI-H186 (cervical carcinoma) (provided by H. Löhrke, German Cancer Research Center, Heidelberg); Ad 211 (pleomorphic adenoma) [34]; NB-4 (promyelocytic leukemia) [35]; FTC133 and FTC238 (follicular thyroid carcinoma) [36]; TPC-1 (papillary thyroid carcinoma) [37]; Li14 (lipoma) [38]; FRO (anaplastic thyroid carcinoma) [39]; WRO (follicular carcinoma) [40]; supT1 (T cell lymphoblastic lymphoma) [41]; HCT116 (colon carcinoma) [42]. They were cultured in RPMI 1640, TC 199 or McCoy's 5A medium supplemented with 10% or 20% fetal bovine serum and 2% penicillin/streptomycin (all Invitrogen, Karlsruhe, Germany). All cells were incubated at 37°C in a humidified incubator with 5% CO_2_. Sample MM 31 was taken during surgery, immediately frozen in liquid nitrogen, and stored at −80°C for RNA isolation.

### RNA-Isolation

Total RNA was purified from cell lines and the tissue sample according to the “RNeasy mini protocol for isolation of total RNA from heart, muscle and skin tissue” (Qiagen, Hilden, Germany) including on-column DNase I digest and homogenisation with QIAshredder©. Following quantification, 5 µg RNA have been digested a second time with DNaseI (6.75 U) for 15 min at room temperature and a cleanup according to the RNeasy mini protocol was performed to remove possible contaminating DNA completely.

### Chromatin Immunoprecipitation

Approximately 1×10^7^ HCT116 cells were harvested with TrypLE Express (Invitrogen, Karlsruhe, Germany) and the cell suspension was transferred into a sterile tube filled with McCoy's 5A medium. Proteins were crosslinked to the DNA using a final concentration of 1% formaldehyde for 10 min at room temperature. The formaldehyde was quenched with 0.125 M glycine (final concentration). After centrifugation the cell pellet was rinsed with an ice-cold PBS/AEBSF solution and then suspended in ChIP Lysis Buffer (Santa Cruz, Heidelberg, Germany). The sample was incubated on ice for 5 min and the pellet was rinsed with an ice-cold PBS/AEBSF solution again. For sonication, the pellet was suspended in 300 µl ChIP Lysis Buffer High Salt (Santa Cruz, Heidelberg, Germany). Fragmentation of the DNA was performed on ice, first to isolate and break down the nuclei and then to fragment the DNA (size 200–500 nucleotides). The parameters were 10 s pulse on and 20 s pulse off for 37.5 min with a Bandelin sonicator HD 3200 plus (Bandelin, Berlin, Germany). The sheared chromatin was cleared by centrifugation at 4°C (10 min at 10,621×g).

Magnetic Dynabeads protein G (Invitrogen, Karlsruhe, Germany) were prepared before usage following the manufacturer's instruction. To reduce the background signal a preclearing step was performed. 100 µl beads were added to the sample and the suspension was incubated for 30 min at 4°C with rotation. The supernatant was transferred and divided into two fractions (IP and NoAb). 4 µg anti-HMGA2 antibody (Santa Cruz, Heidelberg, Germany) were added to the IP sample, the fraction without antibody (NoAb) served as a negative control. Both fractions were incubated over night at 4°C on a rotator. To avoid unspecific interactions between DNA and beads, Dynabeads protein G were rotated with 22.2 µg salmon sperm DNA for 30 min at 4°C before use. After this second preclearing step, the IP and NoAb fractions were incubated on a rotator for 2 h at 4°C each with 50 µl of the blocked Dynabead suspension. The immune complexes were washed two times with 1 ml ChIP Lysis Buffer, four times with ChIP Lysis Buffer High Salt and ChIP Wash Buffer (Santa Cruz) and once with 1× TE buffer (10 mM Tris base, 1 mM EDTA). All washing steps were carried out at 4°C. To reverse crosslinks the Dynabeads protein G were suspended in 150 µl SDS elution buffer (1% SDS, 0.1 M NaHCO_3_) and incubated in a shaking water bath for 2 h at 67°C. The supernatants were transferred into new 2 ml plastic tubes and incubated with 5 µg Proteinase K (Qiagen, Hilden, Germany) for another 2 h at 67°C. To avoid precipitation during the DNA isolation the samples were diluted 1∶2 with H_2_O. The DNA was isolated using the QIAquick PCR Purification Kit (Qiagen, Hilden, Germany) following the manufacturer's instructions.

### SDS-PAGE and Western Blotting

The protein concentration was measured with the BCA Protein Assay Kit (Pierce, Bonn, Germany) 15 µg of protein obtained from each sample were used for SDS-PAGE in a X-Cell Sure Lock Mini-Cell apparatus (Invitrogen, Karlsruhe, Germany) and transferred to a nitrocellulose membrane with the Fastblot 33 system (Biometra, Göttingen, Germany). The membrane was blocked with 5% BSA over night and incubated with rabbit polyclonal anti-HMGA2 antibody (1∶3000, Biocheck, Foster City, USA) and mouse monoclonal anti-β-actin (1∶7500, Novus Biologicals, Cambridge, United Kingdom) for one hour. Second antibodies were alkaline phosphatase-bovine anti-rabbit IgG (1∶3750, Sante Cruz, Heidelberg, Germany) and alkaline phosphatase-goat anti-mouse IgG (1∶7500, Invitrogen, Karlsruhe, Germany). The detection of β-actin was used as an internal control to confirm equivalent total protein loading. Relative HMGA2 protein expression was determined by band intensities with the ImageJ program.

For determination of protein expression in the ChIP samples, supernatants of samples after DNA-protein-antibody-bead-complex formation (IP and NoAb) and samples before Proteinase K digestion (IP and NoAb) were taken. Proteins were separated by SDS-PAGE as described by Laemmli [43] using the Minigel-System Protean II and transferred to a polyvinyl difluoride membrane using the Mini-Transblot System (Biorad, Munich, Germany). The membrane was blocked with TBS-T buffer (10 mM Tris-HCl, 150 mM NaCl, 1% Tween-20) containing 5% skimmed milk and incubated with rabbit polyclonal anti-HMGA2 antibody (1∶800, Santa Cruz, Heidelberg, Germany) for one hour. The second antibody-step was performed with the alkaline phosphatase-goat anti-rabbit IgG (1∶5000, Invitrogen, Karlsruhe, Germany) and bands were visualized by adding BCIP/NBT substrate (Roche Diagnostics, Mannheim, Germany).

### Real-time PCR

All real-time PCRs were run on an ABI Prism 7300 Sequence Detection System (Applied Biosystems, Darmstadt, Germany). For Quantification of *HMGA2* 250 ng total RNA were reverse transcribed with 200 units of M-MLV Reverse Transcriptase (Invitrogen, Karlsruhe, Germany) and 150 ng random hexamers (Fisher Scientific, Schwerte, Germany) according to the manufacturer's instructions. The relative quantification method was carried out using 18S rRNA as endogenous control (forward primer 5′-GGA TCC ATT GGA GGG CAA GT-3′; reverse primer 5′-AAT ATA CGC TAT TGG AGC TGG AAT TAC-3′ and probe 5′-6-FAM-TGC CAG CAG CCG C-MGB-3′) [44]. The samples were diluted (1∶10) for the endogenous control due to higher expression level of 18S rRNA. *HMGA2* (Assay Hs00171569_m1, Applied Biosystems) and 18S rRNA expression analyses were performed in triplicate in a total volume of 20 µl using 2 µl of each cDNA corresponding to 25 ng of total RNA. The expression of the endogenous control 18S rRNA showed only a slight variation in all the samples, the mean Ct value was 8.17±0.21.

IP fragments were analyzed in triplicates starting with 3 µl of template DNA. The enrichment of DNA in the samples (IP, NoAb) was determined by amplification of *GAPDH*. The sequences for *GAPDH* were 5_-6-FAM-AAA GAG CTA GGA AGG ACA GGC AAC TTG GC-TAMRA-3_ for the fluorescent probe, 5_-CCC CAC ACA CAT GCA CTT ACC-3_ for the forward primer, and 5_-CCT AGT CCC AGG GCT TTG ATT-3_ for the reverse primer (Operon, Cologne, Germany). PCR condition were 50°C for 2 min, 95°C for 10 min and 50 cycles of 95°C for 15 s and 60°C for 1 min. Results were calculated by subtracting the Ct-value of the sample from the corresponding NoAb control, followed by 2^(NoAb-IP)^ to evaluate the *x*-fold higher amount of starting material of the sample applied in the real-time PCR.

### Cloning of immunoprecipitated products

The ChIP-generated DNA fragments were A-tailed and ligated into the pGEM©-T easy vector (Promega, Mannheim, Germany) with T4 ligase at 4°C over night. The transformation was carried out according to the manufacturer's protocol with 100 µl *Escherischia coli* DH5α competent cells. 150 µl respectively 200 µl of the transformation culture were plated onto AIX-plates and incubated over night at 37°C. Plasmid DNA was isolated with the QIAprep Spin Miniprep Kit (Qiagen, Hilden, Germany) following the manufacturer's protocol.

### *In silico* data analysis

Clones were sequenced by Eurofins MWG Operon (Ebersberg, Germany). After revising with the Lasergene software, a BLAST search of the human genome database at NCBI was performed to locate the sequences.

For identifying the consensus sequences in the human genomic sequences (NCBI refseq build 36) Perl (www.perl.org) with implemented Bio-Perl Modules [45] has been used. The possible genomic binding sequences have been identified by pattern matching. Specific sequences have been analyzed also using BLAST [46] adjusted to short sequences (Program = blastn, Word size = 7, Expect Value = 100, Filter = disabled). For statistical analysis the statistics software R (www.r-project.org) has been used. The sequence logo was generated by WebLogo (http://weblogo.berkeley.edu/logo.cgi).

## Supporting Information

Figure S1 **Scheme of the HMGA2 chromatin immunoprecipitation cloning procedure.** Cells were crosslinked with formaldehyde to preserve the structure of chromatin and proteins. After lysis and sonication the fragmented DNA was immunoprecipitated with a HMGA2-antibody. For Western Blot analysis aliquots were taken after immunoprecipitation. Crosslinks were reversed in the rest of the samples, DNA was eluted and enrichment of ChIP DNA fragments was measured by real-time PCR. ChIP DNA fragments of the remaining samples were cloned into a vector, sequenced and analyzed.
(TIF)

Table S1 Sequences of the cloned fragments.
(XLS)
