# Supplementary figures and images for: Chromatin Immunoprecipitation to Analyze DNA Binding Sites of HMGA2
Source: PLoS One. 2011 Apr 14;6(4):e18837. doi: 10.1371/journal.pone.0018837 (PMC3077414; doi:10.1371/journal.pone.0018837)

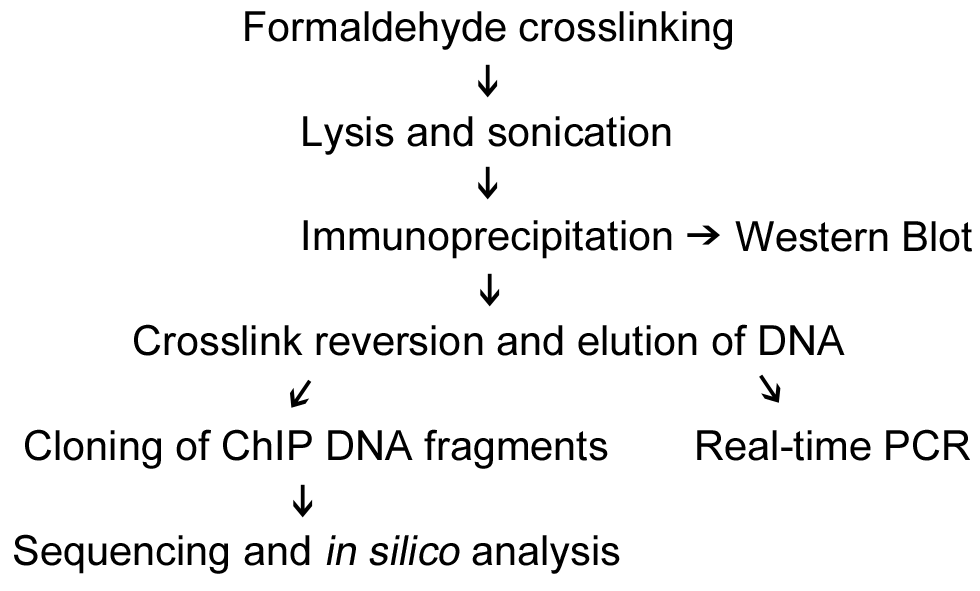

Supplement: Figure S1 — Scheme of the HMGA2 chromatin immunoprecipitation cloning procedure. Cells were crosslinked with formaldehyde to preserve the structure of chromatin and proteins. After lysis and sonication the fragmented DNA was immunoprecipitated with a HMGA2-antibody. For Western Blot analysis aliquots were taken after immunoprecipitation. Crosslinks were reversed in the rest of the samples, DNA was eluted and enrichment of ChIP DNA fragments was measured by real-time PCR. ChIP DNA fragments of the remaining samples were cloned into a vector, sequenced and analyzed. (TIF) [file pone.0018837.s001.tif]
